# Your uncertainty, your probability, your decision

**DOI:** 10.3389/fgene.2013.00148

**Published:** 2013-08-07

**Authors:** Alex Biedermann

**Affiliations:** Faculty of Law and Criminal Justice, School of Criminal Justice, University of LausanneLausanne, Switzerland

In 1973, in his foreword for de Finetti's book “Theory of Probability, A critical introductory treatment”, Professor Lindley wrote that “(…) every now and again delightful ideas spring to view; the idea that we shall all be Bayesian by 2020 (…). But, as I said, this is a book about life” (Finetti, 1974, ix). The two strains of idea, that we should all use probability to approach uncertainty, and that research that has been done on this topic in the twentieth century has the potential to “(…) affect the activities of many people and ultimately all of us” (Lindley, 2006, xiv), is also central to his current book “Understanding Uncertainty” Lindley (2006). Thirty years ago, the year 2020 may have appeared far ahead in the future, but today we can see 2020 showing up at the horizon. So, the question is, where do we stand with our understanding of probability and uncertainty? This question is one of concern for Professor Lindley, as he writes: “(…) I made a discovery. There were people out there, like politicians, journalists, lawyers, and managers, who were, in my opinion, making mistakes; mistakes that could have been avoided had they known the answers to the questions pondered in my ivory tower” (Lindley, 2006, xiv). These words were not intended to be critical. Rather, they express the view that it is up to academics to communicate and “(…) explain in terms that motivated, lay persons can understand, some of the discoveries made in academe, and why they are of importance and value to them, so that they might use the results in their lives” (Lindley, 2006, xiv).

The author makes every effort to achieve this goal. The book is written with exceptional clarity, and the arguments are presented in a way that directly addresses the reader “(…) conveniently called ‘you’ (…)” (Lindley, 2006, 1, 2). The “you” is a stylistic choice that places the author directly in line with other influential authors who hold the so-called subjectivistic interpretation of probability theory^1^. This reinforces the author's intention to place the readers in the center of the argument: in fact, he notes “[t]his book is for you, whoever you are” (Lindley, 2006, 2). This intention stems directly from one of the book's main messages: that probability is inevitable.

The book is not primarily about the *calculus* of probability (indeed, mathematics are kept to a strict minimum); it is about the very *meaning* of probability—how probability ought to be understood in order to deal with uncertainty. On this latter point, Professor Lindley is uncompromisingly clear and, at the same time, draws yet another parallel to de Finetti's two-volume work on probability Finetti (1974, 1975): probability is the measure for strength of belief, but probability *does not exist* in the sense of being a property of the outside world. On a first view, specialized readers of this Frontiers journal may find this proposition all too general, or even inappropriate. But it is not, for several reasons.

Indeed, it is common for forensic science commentators to use the abstract expression *the probability* for some event. Also, scientists may feel or object that they could not ascertain a particular number, only so-called upper and lower probabilities. However, uncertainty about a given proposition may vary between persons, because their extent of knowledge may differ, hence the reason why it is more appropriate to refer to *your probability*, ours or anybody's. Moreover, Professor Lindley presents us with persuasive argument that probability is given by a single number.

Further examples that point out the relevance of this book for forensic specialists can readily be found. Suffice to note that, often, probability and likelihood are used as synonyms. Similarly, probability is often equated with frequency. Here the author emphasizes that these ideas are inappropriate. These terms have very distinct and precise meanings, and it goes without saying that these distinctions have a potential to help clarify and improve the rigor of forensic science communications. So, if you think or have always thought that you can pass from frequency to a belief in a straightforward way, then you might confuse a notion that refers to data with one that refers to belief, and this book will show you that passing from one notion to the other is *not* straightforward.

This book is about its readers' uncertainty, and the book's title “Understanding Uncertainty” essentially “(…) means knowing the three rules of probability” (Lindley, 2006, 66). This topic deals with how one's beliefs should be organized, but there is a further important subject that the book brings to the attention of the reader: the *use* of beliefs in action. How might one decide between different courses of action? This question moves the discussion from uncertainty to possible consequences of actions taken under uncertainty, the expression—called utility—of the desirability of these consequences, and the maximization of expected utility as a basis for action. Currently, forensic and legal writings draw little attention to thoughts on how to extend the view from probability and uncertainty to analyzing how to decide sensibly between possible actions. Yet, questions of decision making abound in forensic science practice Taroni et al. (2005) (e.g., “should DNA profiling analyses be performed in this case or not?”) and, ultimately, in court Kaye (1999).

To attempt a review of a book of an eminent academic such as Professor Lindley is both difficult and daring for a generalist. The “review” here thus is not written from a position that claims authority—rather, it is a tribute to a work that has the value of inspiring the practice of forensic science to serve society better, even though the general theme requires much challenging thought. The words with which Professor Lindley described de Finetti's work, “[t]he author has words of wisdom to say about many things and the wisdom often only appears after reflection” (Finetti, 1974, ix), clearly apply also for Lindley's own work “Understanding Uncertainty.”

## Footnotes

^1^Indeed, in de Finetti's treatise we find the following parallel: “Let us introduce right away the use of ‘You,’ following Good (Savage uses ‘Thou’).” (Finetti, 1974, 27).
